# Reversible left ventricular noncompaction caused by hypertensive hydrocephalus: a pediatric case report

**DOI:** 10.1186/s12887-021-02680-6

**Published:** 2021-04-28

**Authors:** Riko Kato, Hiromichi Taneichi, Shinya Takarada, Mako Okabe, Nariaki Miyao, Hideyuki Nakaoka, Keijiro Ibuki, Sayaka Ozawa, Yuichi Adachi, Naoki Yoshimura, Kazuyoshi Saito, Fukiko Ichida, Keiichi Hirono

**Affiliations:** 1 Department of Pediatrics, Faculty of Medicine, University of Toyama, 2630 Sugitani, Toyama, 930-0194 Japan; 2 First Department of Surgery, Faculty of Medicine, University of Toyama, Toyama, Japan; 3 Department of Pediatrics, Fujita Health University, Toyoake city, Aichi Japan; 4 Department of Pediatrics, International University of Health and Welfare, Tokyo, Japan

**Keywords:** Left ventricular noncompaction, Hypertensive hydrocephalus

## Abstract

**Background:**

Left ventricular noncompaction cardiomyopathy (LVNC) is characterized by prominent ventricular trabeculations on cardiovascular imaging. Acquired reversible LVNC has not been reported in pediatrics without a genetic background.

**Case presentation:**

A 9-year-old girl with a ventriculoperitoneal (VP) shunt for neonatal posthemorrhagic hydrocephalus was referred due to exacerbation of hydrocephalus caused by VP shunt dysfunction. Transthoracic echocardiography (TTE) revealed depressed left ventricular (LV) systolic function and thick prominent trabeculae in the LV, predominantly in the apex, suggesting LVNC. Following treatment with extraventricular drainage for hydrocephalus, prominent trabeculation of the LV was diminished on TTE within 3 months. Genetic testing using next-generation sequencing was performed, and no significant variants were identified.

**Conclusions:**

We revealed for the first time a pediatric case of reversible LVNC without genetic predisposition. This case report provides valuable information on the pathogenesis of acquired LVNC and suggests that detailed evaluation is required to elucidate the diagnosis of this wide spectrum of etiologic–pathogenetic disorders.

**Supplementary Information:**

The online version contains supplementary material available at 10.1186/s12887-021-02680-6.

## Background

Left ventricular noncompaction cardiomyopathy (LVNC) is characterized by prominent ventricular trabeculations on cardiovascular imaging. Acquired reversible LVNC has been recently reported in adults but not in children [1]. Here, we report a pediatric case of acquired reversible LVNC that resulted from hypertensive hydrocephalus without a genetic background.

## Case presentation

**Fig. 1:**
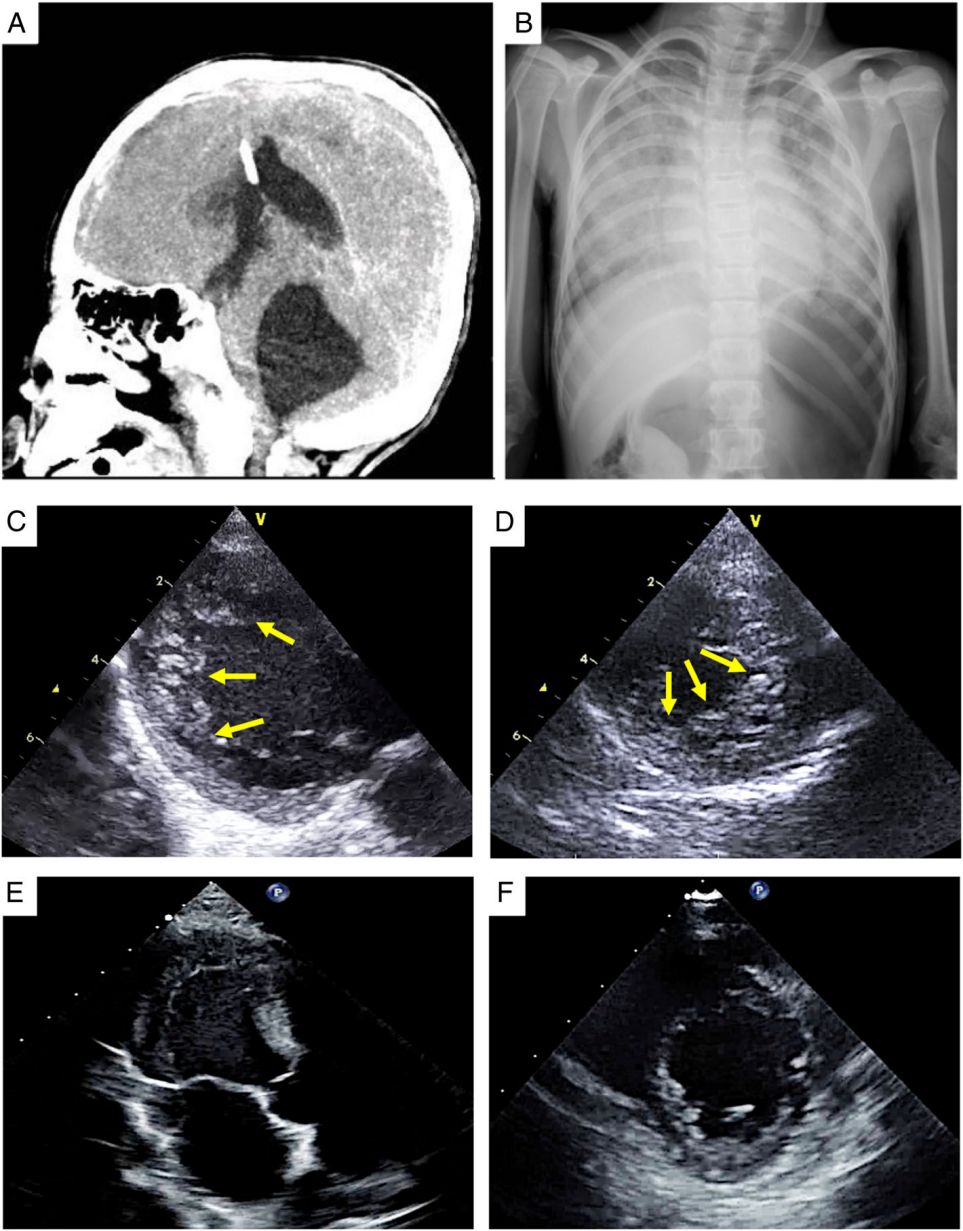
Head CT image (**a**), chest radiograph (**b**), and TTE images showing prominent trabeculations in the LV (**c** and **d**), subsequently, trabeculation no longer observable (**e** and **f**). TTE depicts heavy prominent trabeculae in the LV, predominantly in the apex, in the parasternal long axis view (**c**) and short axis view (**d**) on the 3rd day of illness. After 3 months, prominent trabeculation of the LV was no longer observable in the parasternal long axis view (**e**) and short axis view (**f**). The arrow indicates prominent trabeculations

A 9-year-old girl with a ventriculoperitoneal (VP) shunt for neonatal posthemorrhagic hydrocephalus was referred to our hospital due to vomiting and headache on the 3rd day of illness. She had no family history of cardiomyopathy or neuromuscular diseases. Soon after, she developed convulsions, consciousness disturbances, and acute respiratory failure and subsequently required intubation. Head computed tomography revealed that the lateral and fourth ventricles were expanded, and the patient was diagnosed with exacerbation of hydrocephalus caused by VP shunt dysfunction (Fig. 1a and Supplemental figure S1). Chest radiography exhibited marked bilateral pulmonary edema (Fig. 1b), and transthoracic echocardiography (TTE) revealed depressed left ventricular (LV) systolic function (left ventricular ejection = 25%). TTE also showed thick prominent trabeculae in the LV, predominantly in the apex, suggesting LVNC (Fig. 1c and d). Previously, this patient was followed for a small atrial septal defect (3 mm of diameter) without volume overload of right atrium and ventricle up to the age of 4 years, confirmed spontaneous closure; however, LVNC had not been observed. Electrocardiography (ECG) showed a negative T wave and QT interval prolongation (corrected QT value by Bazett’s formula, 0.529) (Supplemental figure S2) along with marked elevation of cardiac biomarker levels (troponin, 2551.4 ng/dL; N-terminal-pro B-type natriuretic peptide, 13,115 pg/mL). Simultaneously, catecholamine levels (adrenaline, noradrenaline, and dopamine) were markedly elevated (1230 pg/mL, 557 pg/mL, and 164,707 pg/mL, respectively). Dopamine and dobutamine (both five μg/kg/minute), and olprinone (0.1 μg/kg/minute) were infused for 3 days and 7 days, respectively. Following treatment with extraventricular drainage for hydrocephalus, the patient recovered rapidly from respiratory failure. She underwent extubation on the 7th day of illness, and VP shunt replacement was performed on the 12th day. Meanwhile, the cardiac biomarker and catecholamine levels decreased rapidly. She was discharged from the hospital on the 21st day of illness without any complications. After 3 months, prominent trabeculation of the LV was no longer observable on TTE (Fig. 1e and f), and significant improvement was found in ST elevation and giant negative T wave on ECG (Supplemental figure S2). Genetic testing using next-generation sequencing with a cardiomyopathy-associated gene panel, which included 182 genes, was performed (Supplemental tables 1 and 2), and no significant variants were identified.

## Discussion and conclusions

To the best of our knowledge, this is the first pediatric case of reversible and transient LVNC requiring intensive care in the perioperative period of hypertensive hydrocephalus that developed without genetic predisposition.

A previous study has shown that transient LVNC with hypertensive hydrocephalus complicated an intracranial neoplasm [2]. During pressure overload of the LV, effective treatment of hypertension or critical aortic stenosis was associated with a change in LV morphology and decrease in LV mass [3]. Because LVNC is frequently associated with systolic dysfunction, additional stress to the myocardium may trigger the deterioration of systolic function. Our case showed that the occurrence of catecholamine stress during progressive hydrocephalus might cause trabeculations to become apparent on echocardiography. Catecholamine surges can trigger coronary microcirculation dysfunction and impair LV contractility in predisposed subjects as well as neurogenic LV stunning [4, 5]. A sympathetic nervous system storm also induces the production of adrenal catecholamines, which exert receptor-operated calcium channels, leading to an acute impairment of LV function and to coronary microvascular constriction [4–6]. For that reason, we speculated that the LVNC was associated with catecholamine surges and exacerbation of hydrocephalus in this patient. Takotsubo cardiomyopathy is mostly induced by physical or emotional stress, characterized by anginal chest pain, ECG abnormalities resembling myocardial infarction, but normal coronary angiography, typically akinesia or hypokinesia of the apex and the left midventricular myocardium, severely reduced systolic function, and complete regression of the ECG and echocardiographic abnormalities [7, 8]. Our case was compatible in some parts of Takotsubo cardiomyopathy; 1) induced by physical stress, 2) ECG abnormalities, 3) reduced systolic function, and 4) reversible ECG and echocardiographic findings. However, not focal hypokinesis of LV but prominent trabeculations were observed in our patient. Thus, we concluded that this patient had a transient LVNC.

The pathogenesis of LVNC has not yet been fully elucidated, and it is unlikely to be a result of a single developmental mechanism. Recent studies have reported that numerous genetic disorders are associated with LVNC, including sarcomere and Z-disk gene mutations, mitochondrial disorders, and ion channel gene mutations. However, similar cases suggesting acquired LVNC have also been noted in adults such as athletes or in conditions such as sickle cell anemia, pregnancy, myopathies, and chronic renal failure [1, 9]. These previous adult cases did not show the existence of a genetic background, consistent with our case.

In conclusion, we revealed for the first time a pediatric case of reversible LVNC without genetic predisposition. Our case study also demonstrated that a detailed evaluation of the diagnosis of a wide spectrum of etiologic–pathogenetic disorders from arrested maturation of LV trabeculae during embryogenesis to acquired pathogenetic mechanisms, including hemodynamic, genetic, or epigenetic factors, is important and could lead to further understanding of LVNC.

## Supplementary Information

**Additional file 1: Figure S1.** Head CT images before (A) and after (B) exacerbation of hydrocephalus. **Figure S2.** Serial ECG changes after admission. **Table S1.** List of 182 analyzed genes of NGS. **Table S2.** Silico predictive algorithms used in the study.

## Data Availability

The authors confirm that the data supporting the findings of this study are available within the article and its supplementary materials.

## Abbreviations

LV: Left ventricle
LVNC: Left ventricular noncompaction cardiomyopathy
VP: Ventriculoperitoneal
TTE: Transthoracic echocardiography
