# Supplementary material for: Reversible left ventricular noncompaction caused by hypertensive hydrocephalus: a pediatric case report
Source: BMC Pediatr. 2021 Apr 28;21:205. doi: 10.1186/s12887-021-02680-6 (PMC8080348; doi:10.1186/s12887-021-02680-6)
Supplement: Supplementary file 1 — Additional file 1: Figure S1. Head CT images before (A) and after (B) exacerbation of hydrocephalus. Figure S2. Serial ECG changes after admission. Table S1. List of 182 analyzed genes of NGS. Table S2. Silico predictive algorithms used in the study. [file 12887_2021_2680_MOESM1_ESM.docx]

**Supplementary Appendix**

**Methods**

**Mutation screening**

After obtaining informed consent from a patient’s parents, DNA was isolated from a sample of whole blood or heart tissue. NGS of 182 cardiac disorder-related genes associated with cardiomyopathies and channelopathies (Table S1) was performed using an Ion PGM System (Life Technologies, Carlsbad, CA, USA).

This custom panel utilized two separate PCR primer pools, yielding a total of 1,870 amplicons and used to generate target amplicon libraries. Genomic DNA samples were PCR-amplified using the custom panel and an Ion AmpliSeq Library Kit v2.0 (Life Technologies, Carlsbad, CA, USA). Individual samples were labeled using an Ion Xpress Barcode Adapters Kit (Life Technologies) and then pooled at equimolar concentrations. Emulsion PCR and ion sphere particle (ISP) enrichment were performed using the Ion PGM Hi-Q OT2 Kit (Life Technologies), according to the manufacturer’s instructions. ISPs were loaded onto a 316 chip and sequenced using an Ion PGM Hi-Q Sequencing Kit (Life Technologies).

**Sanger sequencing**

For all candidate pathogenic variants that passed these selection criteria, Sanger sequencing was used to validate the NGS results. For this, the nucleotide sequences of amplified fragments were analyzed by direct sequencing in both directions by using the BigDye Terminator v3.1 Cycle Sequencing Kit (Applied Biosystems, Foster City, CA) and sequence analysis was performed using an ABI 3130xl automated sequencer (Applied Biosystems).

**Data analysis and variant classification**

Torrent Suite and Ion Reporter Software 5.0 (Life Technologies) were used to perform primary, secondary, and tertiary analyses, including optimized signal processing, base calling, sequence alignment, and variant analysis.

The allelic frequency of all detected variants was determined using the Exome Aggregation Consortium (ExAC) database and Human Genetic Variation Database (HGVD), which contain data of 1,208 Japanese individuals. All variants with a minor allelic frequency of ≥0.005 in the ExAC and HGVD populations were excluded. To evaluate the pathogenicity of the remaining variants, we utilized seven different *in silico* predictive algorithms: FATHMM, SIFT, PROVEAN, Align GVGD, MutationTaster2, PolyPhen-2, and CADD (Table S2). Variants predicted to be deleterious or pathogenic by at least five of the seven *in silico* algorithms were considered likely pathogenic.

**Figure S1. Head CT images before (A) and after (B) exacerbation of hydrocephalus.**

**
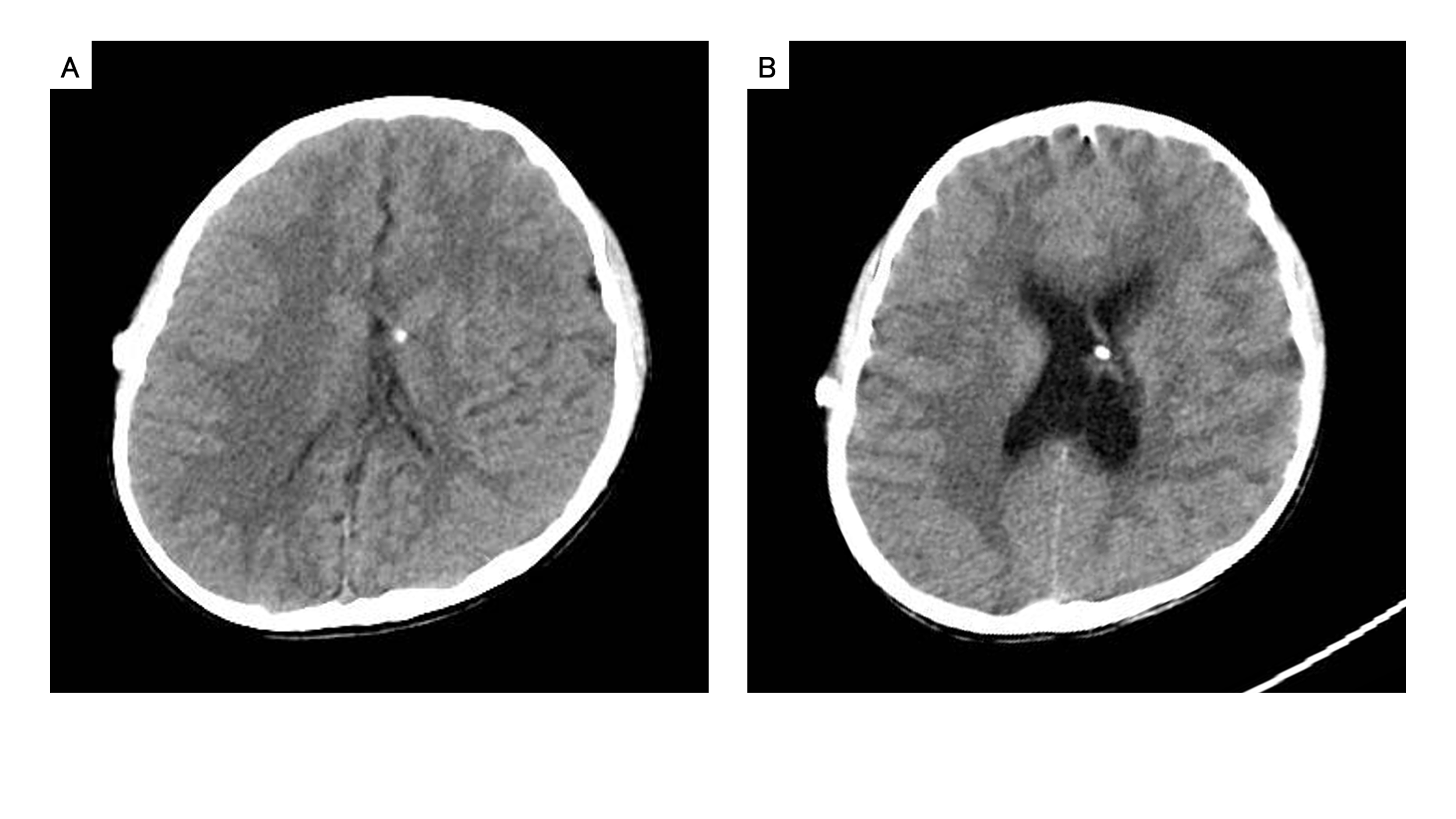
**

**Figure S2. Serial ECG changes after admission.**

**
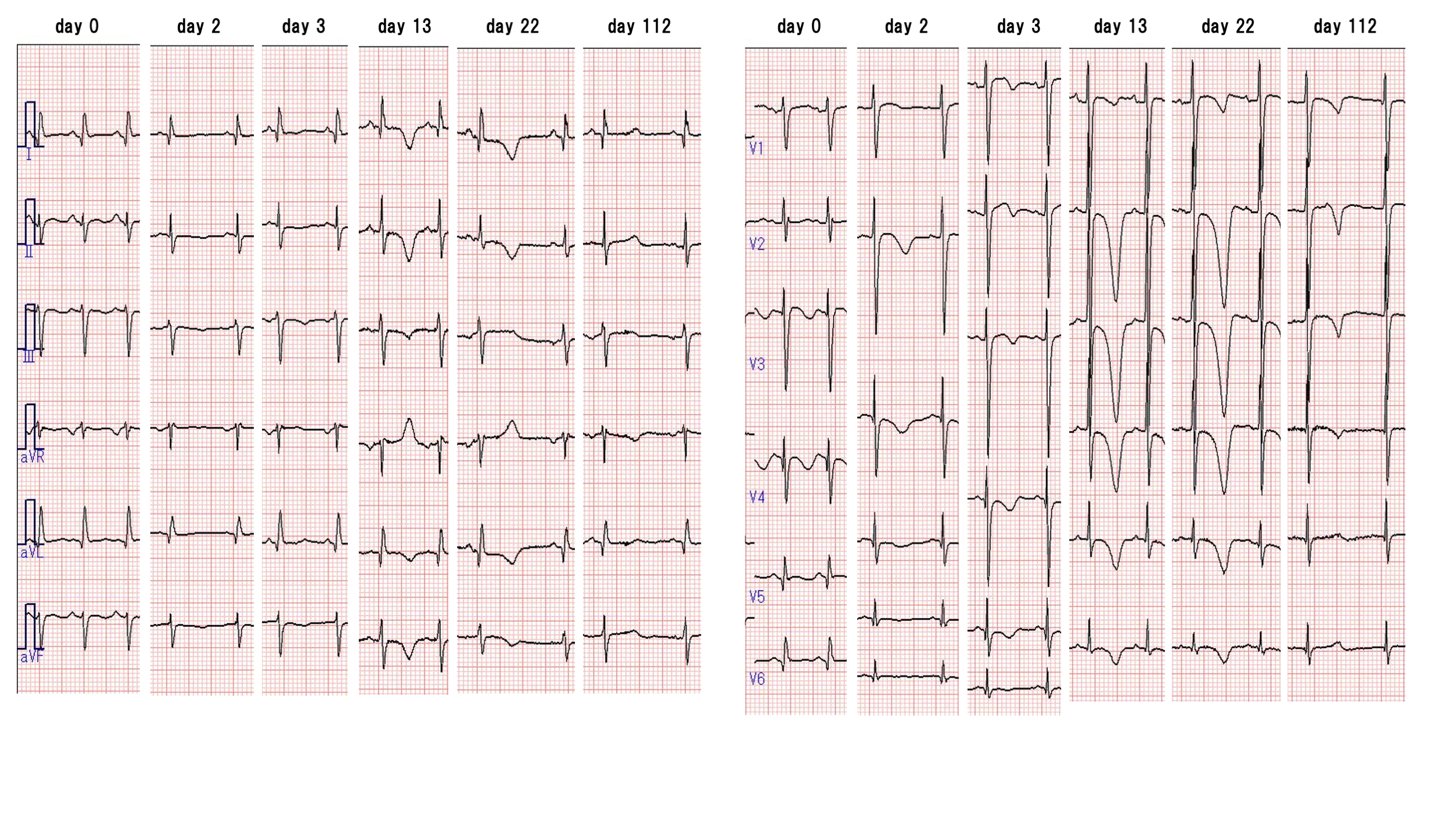
**

**Table S1. List of 182 analyzed genes of NGS.**

| Gene | Chromosome | NCBI Reference Sequence: | Sequence : (Start..End) |  |
| --- | --- | --- | --- | --- |
| *ABCC9* | 12p12.1 | NG_012819.1 | NC_000012.11 (21950323..22094797, complement) | http://www.ncbi.nlm.nih.gov/gene/10060 |
| *ACAD9* | 3q21.3 | NG_017064.1 | NC_000003.12 (128879490..128913114) | <https://www.ncbi.nlm.nih.gov/gene/28976> |
| *ACADVL* | 17p13.1 | NG_007975.1 | NC_000017.11 (7217125..7225267) | <https://www.ncbi.nlm.nih.gov/gene/37> |
| *ACTC1* | 15q14 | NG_007553.1 | NC_000015.9 (35080297..35087927, complement) | http://www.ncbi.nlm.nih.gov/gene/70 |
| *ACTN2* | 1q42-q43 | NG_009081.1 | NC_000001.10 (236849754..236927931) | http://www.ncbi.nlm.nih.gov/gene/88 |
| *ADAMTS1* | 21q21.3 | NC_000021.9 | NC_000021.9 (26836287..26845409, complement) | https://www.ncbi.nlm.nih.gov/gene/9510 |
| *ADAMTS9* | 3p14.1 | NC_000003.12 | NC_000003.12 (64515654..64688000, complement) | <https://www.ncbi.nlm.nih.gov/gene/56999> |
| *ADCK3* | 1q42.13 | NG_012825.2 | NC_000001.11 (226939339..226987545) | <https://www.ncbi.nlm.nih.gov/gene/56997> |
| *AKAP9* | 7q21-q22 | NG_011623.1 | NC_000007.13 (91570181..91739987 | http://www.ncbi.nlm.nih.gov/gene/10142 |
| *AMPD1* | 1p13.2 | NG_008012.1 | NC_000001.11 (114673098..114695618, complement) | <https://www.ncbi.nlm.nih.gov/gene/270> |
| *ANK2* | 4q25-q27 | NG_009006.2 | NC_000004.11 (113739239..114304896) | http://www.ncbi.nlm.nih.gov/gene/287 |
| *ARFGEF2* | 20q13.13 | NG_011490.1 | NC_000020.11 (48921721..49036693) | <https://www.ncbi.nlm.nih.gov/gene/10564> |
| *BAG3* | 10q25.2-q26.2 | NG_016125.1 | NC_000010.10 (121410859..121437331) | http://www.ncbi.nlm.nih.gov/gene/9531 |
| *BMP10* | 2p13.3 | NG_032117.1 | NC_000002.12 (68860916..68871517, complement) | <https://www.ncbi.nlm.nih.gov/gene/27302> |
| *BMPR1A* | 10q22.3 | NG_009362.1 | NC_000010.10 (88516396..88684945) | http://www.ncbi.nlm.nih.gov/gene/657 |
| *BOLA3* | 2p13.1 | NG_031910.1 | NC_000002.12 (74135401..74147912, complement) | <https://www.ncbi.nlm.nih.gov/gene/388962> |
| *BRAF* | 7q34 | NG_007873.3 | NC_000007.14 (140719327..140924928, complement) | <https://www.ncbi.nlm.nih.gov/gene/673> |
| *C10orf2* | 10q24.31 | NG_012624.1 | NC_000010.11 (100987527..100994403) | <https://www.ncbi.nlm.nih.gov/gene/56652> |
| *CACNA1C* | 12p13.3 | NG_008801.2 | NC_000012.11 (2079952..2807115) | http://www.ncbi.nlm.nih.gov/gene/775 |
| *CACNA2D1* | 7q21.11 | NC_000007.14 | NC_000007.14 (81946444..82443806, complement) | <https://www.ncbi.nlm.nih.gov/gene/781> |
| *CACNB2* | 10p12 | NG_016195.1 | NC_000010.10 (18429373..18830688) | http://www.ncbi.nlm.nih.gov/gene/783 |
| *CALR3* | 19p13.11 | NG_031959.2 | NC_000019.9 (16589767..16607015, complement) | http://www.ncbi.nlm.nih.gov/gene/125972 |
| *CAPN3* | 15q15.1 | NG_008660.1 | NC_000015.9 (42646545..42704515) | http://www.ncbi.nlm.nih.gov/gene/825 |
| *CASQ2* | 1p13.1 | NG_008802.1 | NC_000001.11 (115700003..115768805, complement) | <https://www.ncbi.nlm.nih.gov/gene/845> |
| *CASZ1* | 1p36.22 | NC_000001.11 | NC_000001.11 (10636604..10796676, complement) | <https://www.ncbi.nlm.nih.gov/gene/54897> |
| *CAV3* | 3p25 | NG_008797.2 | NC_000003.11 (8775486..8788451) | http://www.ncbi.nlm.nih.gov/gene/859 |
| *CDKN1C* | 11p15.4 | NG_008022.1 | NC_000011.10 (2883218..2885804, complement) | <https://www.ncbi.nlm.nih.gov/gene/1028> |
| *COL4A1* | 13q34 | NG_011544.1 | NC_000013.10 (110801310..110959496, complement) | http://www.ncbi.nlm.nih.gov/gene/1282 |
| *COL7A1* | 3p21.31 | NG_007065.1 | NC_000003.12 (48564073..48595302, complement) | <https://www.ncbi.nlm.nih.gov/gene/1294> |
| *CPT2* | 1p32.3 | NG_008035.1 | NC_000001.11 (53196429..53214197) | <https://www.ncbi.nlm.nih.gov/gene/1376> |
| *CSRP3* | 11p15.1 | NG_011932.2 | NC_000011.10 (19182030..19210571, complement) | <https://www.ncbi.nlm.nih.gov/gene/8048> |
| *CTNNA3* | 10q21.3 | NG_034072.1 | NC_000010.11 (65912518..67696217, complement) | <https://www.ncbi.nlm.nih.gov/gene/29119> |
| *DAAM1* | 14q23.1 | NG_047127.1 | NC_000014.9 (59188657..59371405) | <https://www.ncbi.nlm.nih.gov/gene/23002> |
| *DAAM2* | 6p21.2 | NC_000006.12 | NC_000006.12 (39792366..39904877) | <https://www.ncbi.nlm.nih.gov/gene/23500> |
| *DES* | 2q35 | NG_008043.1 | NC_000002.11 (220283099..220291461) | http://www.ncbi.nlm.nih.gov/gene/1674 |
| *DMD* | Xp21.2 | NG_012232.1 | NC_000023.10 (31137345..33357726, complement) | http://www.ncbi.nlm.nih.gov/gene/1756 |
| *DMPK* | 19q13.32 | NG_009784.1 | NC_000019.10 (45769709..45782557, complement) | <https://www.ncbi.nlm.nih.gov/gene/1760> |
| *DNAJC19* | 3q26.33 | NG_022933.1 | NC_000003.12 (180983709..180989774, complement) | <https://www.ncbi.nlm.nih.gov/gene/131118> |
| *DSC2* | 18q12.1 | NG_008208.1 | NC_000018.9 (28645938..28682388, complement) | http://www.ncbi.nlm.nih.gov/gene/1824 |
| *DSG2* | 18q12.1 | NG_007072.3 | NC_000018.9 (29078027..29128814) | http://www.ncbi.nlm.nih.gov/gene/1829 |
| *DSP* | 6p24 | NG_008803.1 | NC_000006.11 (7541808..7586946) | http://www.ncbi.nlm.nih.gov/gene/1832 |
| *DTNA* | 18q12.1 | NG_009201.1 | NC_000018.10 (34493290..34891844) | <https://www.ncbi.nlm.nih.gov/gene/1837> |
| *DVL1* | 1p36.33 | NG_008048.1 | NC_000001.11 (1335278..1349142, complement) | <https://www.ncbi.nlm.nih.gov/gene/1855> |
| *EED* | 11q14.2 | NG_029595.1 | NC_000011.10 (86244384..86285420) | <https://www.ncbi.nlm.nih.gov/gene/8726> |
| *ELN* | 7q11.23 | NG_009261.1 | NC_000007.13 (73442119..73484237) | http://www.ncbi.nlm.nih.gov/gene/2006 |
| *EMD* | Xq28 | NG_008677.1 | NC_000023.10 (153607597..153609883) | http://www.ncbi.nlm.nih.gov/gene/2010 |
| *ERBB2* | 17q12 | NG_007503.1 | NC_000017.11 (39688084..39728662) | <https://www.ncbi.nlm.nih.gov/gene/2064> |
| *ERBB4* | 2q34 | NG_011805.1 | NC_000002.12 (211375717..212538628, complement) | <https://www.ncbi.nlm.nih.gov/gene/2066> |
| *EZH2* | 7q36.1 | NG_032043.1 | NC_000007.14 (148807372..148884349, complement) | <https://www.ncbi.nlm.nih.gov/gene/2146> |
| *FBN2* | 5q23.3 | NG_008750.1 | NC_000005.10 (128257909..128538042, complement) | <https://www.ncbi.nlm.nih.gov/gene/2201> |
| *FGF16* | Xq21.1 | NG_034050.1 | NC_000023.11 (77447675..77456522) | <https://www.ncbi.nlm.nih.gov/gene/8823> |
| *FGF9* | 13q12.11 | NG_016272.1 | NC_000013.11 (21671076..21704501) | <https://www.ncbi.nlm.nih.gov/gene/2254> |
| *FGFR1* | 8p11.23 | NG_007729.1 | NC_000008.11 (38411138..38468834, complement) | <https://www.ncbi.nlm.nih.gov/gene/2260> |
| *FGFR2* | 10q26.13 | NG_012449.2 | NC_000010.11 (121478330..121598458, complement) | <https://www.ncbi.nlm.nih.gov/gene/2263> |
| *FKBP1A* | 20p13 | NC_000020.11 | NC_000020.11 (1368977..1393172, complement) | <https://www.ncbi.nlm.nih.gov/gene/2280> |
| *FKBP1B* | 2p23.3 | NC_000002.12 | NC_000002.12 (24033205..24067743) | <https://www.ncbi.nlm.nih.gov/gene/2281> |
| *FKTN* | 9q31.2 | NG_008754.1 | NC_000009.12 (105558117..105655950) | <https://www.ncbi.nlm.nih.gov/gene/2218> |
| *FLNA* | Xq28 | NG_011506.1 | NC_000023.11 (154348532..154374638, complement) | <https://www.ncbi.nlm.nih.gov/gene/2316> |
| *FXN* | 9q21.11 | NG_008845.2 | NC_000009.12 (69035563..69079077) | <https://www.ncbi.nlm.nih.gov/gene/2395> |
| *GAA* | 17q25.2-q25.3 | NG_009822.1 | NC_000017.10 (78075339..78093680) | http://www.ncbi.nlm.nih.gov/gene/2548 |
| *GATA4* | 8p23.1-p22 | NG_008177.1 | NC_000008.10 (11534433..11617510) | http://www.ncbi.nlm.nih.gov/gene/2626 |
| *GBE1* | 3p12.2 | NG_011810.1 | NC_000003.12 (81489699..81761799, complement) | <https://www.ncbi.nlm.nih.gov/gene/2632> |
| *GFRA1* | 10q25.3 | NG_050620.1 | NC_000010.11 (116056925..116273645, complement) | <https://www.ncbi.nlm.nih.gov/gene/2674> |
| *GFRA2* | 8p21.3 | NG_029215.1 | NC_000008.11 (21690403..21789296, complement) | <https://www.ncbi.nlm.nih.gov/gene/2675> |
| *GLA* | Xq22 | NG_007119.1 | NC_000023.10 (100652779..100663001, complement) | http://www.ncbi.nlm.nih.gov/gene/2717 |
| *GPD1L* | 3p22.3 | NG_023375.1 | NC_000003.11 (32148003..32210207) | http://www.ncbi.nlm.nih.gov/gene/23171 |
| *HADHA* | 2p23.3 | NG_007121.1 | NC_000002.12 (26190635..26244726, complement) | <https://www.ncbi.nlm.nih.gov/gene/3030> |
| *HAS2* | 8q24.13 | NC_000008.11 | NC_000008.11 (121613031..121641390, complement) | <https://www.ncbi.nlm.nih.gov/gene/3037> |
| *HBB* | 11p15.4 | NG_059281.1 | NC_000011.10 (5225466..5227071, complement) | <https://www.ncbi.nlm.nih.gov/gene/3043> |
| *HCCS* | Xp22.2 | NG_016460.1 | NC_000023.11 (11111286..11123086) | <https://www.ncbi.nlm.nih.gov/gene/3052> |
| *HCN4* | 15q24.1 | NG_009063.1 | NC_000015.9 (73612200..73661605, complement) | http://www.ncbi.nlm.nih.gov/gene/10021 |
| *HEY2* | 6q22.31 | NC_000006.12 | NC_000006.12 (125747639..125762243) | <https://www.ncbi.nlm.nih.gov/gene/23493> |
| *HMGCL* | 1p36.11 | NG_013061.1 | NC_000001.11 (23801877..23825459, complement) | <https://www.ncbi.nlm.nih.gov/gene/3155> |
| *ITGA7* | 12q13.2 | NG_012343.1 | NC_000012.12 (55684568..55716037, complement) | <https://www.ncbi.nlm.nih.gov/gene/3679> |
| *JARID2* | 6p22.3 | NC_000006.12 | NC_000006.12 (15245975..15522042) | <https://www.ncbi.nlm.nih.gov/gene/3720> |
| *JUP* | 17q21 | NG_009090.2 | NC_000017.10 (39910859..39942964, complement) | http://www.ncbi.nlm.nih.gov/gene/3728 |
| *KCNE1* | 21q22.12 | NG_009091.1 | NC_000021.8 (35790910..35884573, complement) | http://www.ncbi.nlm.nih.gov/gene/3753 |
| *KCNE2* | 21q22.12 | NG_008804.1 | NC_000021.8 (35736323..35743440) | http://www.ncbi.nlm.nih.gov/gene/9992 |
| *KCNE3* | 11q13.4 | NG_011833.1 | NC_000011.9 (74165886..74178600, complement) | http://www.ncbi.nlm.nih.gov/gene/10008 |
| *KCNH2* | 7q36.1 | NG_008916.1 | NC_000007.13 (150642044..150675402, complement) | http://www.ncbi.nlm.nih.gov/gene/3757 |
| *KCNJ2* | 17q24.3 | NG_008798.1 | NC_000017.10 (68164757..68176189) | http://www.ncbi.nlm.nih.gov/gene/3759 |
| *KCNQ1* | 11p15.5 | NG_008935.1 | NC_000011.9 (2466221..2870340) | http://www.ncbi.nlm.nih.gov/gene/3784 |
| *KRAS* | 12p12.1 | NG_007524.1 | NC_000012.11 (25358180..25403870, complement) | http://www.ncbi.nlm.nih.gov/gene/3845 |
| *LAMP2* | Xq24 | NG_007995.1 | NC_000023.10 (119560003..119603204, complement) | http://www.ncbi.nlm.nih.gov/gene/3920 |
| *LDB3* | 10q22.3-q23.2 | NG_008876.1 | NC_000010.10 (88426542..88495829) | http://www.ncbi.nlm.nih.gov/gene/11155 |
| *LMNA* | 1q22 | NG_008692.2 | NC_000001.10 (156052369..156109880) | http://www.ncbi.nlm.nih.gov/gene/4000 |
| *LMX1B* | 9q33.3 | NG_017039.1 | NC_000009.12 (126614443..126701032) | <https://www.ncbi.nlm.nih.gov/gene/4010> |
| *LRPPRC* | 2p21 | NG_008247.1 | NC_000002.12 (43886224..43996005, complement) | <https://www.ncbi.nlm.nih.gov/gene/10128> |
| *MADD* | 11p11.2 | NG_029462.1 | NC_000011.10 (47269376..47330031) | <https://www.ncbi.nlm.nih.gov/gene/8567> |
| *MBL2* | 10q21.1 | NG_008196.1 | NC_000010.11 (52764977..52772847, complement) | <https://www.ncbi.nlm.nih.gov/gene/4153> |
| *MED1* | 17q12 | NG_046996.1 | NC_000017.11 (39404285..39451281, complement) | <https://www.ncbi.nlm.nih.gov/gene/5469> |
| *MEST* | 7q32.2 | NG_009226.1 | NC_000007.14 (130486175..130506297) | <https://www.ncbi.nlm.nih.gov/gene/4232> |
| *MIB1* | 18q11.2 | NG_033272.2 | NC_000018.10 (21740793..21870957) | <https://www.ncbi.nlm.nih.gov/gene/57534> |
| *MIPEP* | 13q12.12 | NG_052977.1 | NC_000013.11 (23730189..23889448, complement) | <https://www.ncbi.nlm.nih.gov/gene/4285> |
| *MLYCD* | 16q23.3 | NG_009079.1 | NC_000016.10 (83899125..83916182) | <https://www.ncbi.nlm.nih.gov/gene/23417> |
| *MMACHC* | 1p34.1 | NG_013378.1 | NC_000001.11 (45500184..45511266) | <https://www.ncbi.nlm.nih.gov/gene/25974> |
| *MRPS22* | 3q23 | NG_012174.1 | NC_000003.12 (139344014..139357129) | <https://www.ncbi.nlm.nih.gov/gene/56945> |
| *MTO1* | 6q13 | NG_032856.1 | NC_000006.12 (73461731..73501456) | <https://www.ncbi.nlm.nih.gov/gene/25821> |
| *MYBPC3* | 11p11.2 | NG_007667.1 | NC_000011.9 (47352957..47374253, complement) | http://www.ncbi.nlm.nih.gov/gene/4607 |
| *MYCN* | 2p24.3 | NG_007457.1 | NC_000002.12 (15940438..15947007) | <https://www.ncbi.nlm.nih.gov/gene/4613> |
| *MYH11* | 16p13.11 | NG_009299.1 | NC_000016.9 (15796992..15950887, complement) | http://www.ncbi.nlm.nih.gov/gene/4629 |
| *MYH6* | 14q12 | NG_023444.1 | NC_000014.8 (23849942..23878836, complement) | http://www.ncbi.nlm.nih.gov/gene/4624 |
| *MYH7* | 14q12 | NG_007884.1 | NC_000014.8 (23881947..23904870, complement) | http://www.ncbi.nlm.nih.gov/gene/4625 |
| *MYH7B* | 20q11.22 | NG_016984.2 | NC_000020.11 (34955835..35002437) | <https://www.ncbi.nlm.nih.gov/gene/57644> |
| *MYL2* | 12q24.11 | NG_007554.1 | NC_000012.11 (111348623..111358404, complement) | http://www.ncbi.nlm.nih.gov/gene/4633 |
| *MYL3* | 3p21.3-p21.2 | NG_007555.2 | NC_000003.11 (46899357..46904973, complement) | http://www.ncbi.nlm.nih.gov/gene/4634 |
| *MYLK* | 3q21 | NG_029111.1 | NC_000003.11 (123331143..123603149, complement) | http://www.ncbi.nlm.nih.gov/gene/4638 |
| *MYOZ2* | 4q26-q27 | NG_029747.1 | NC_000004.11 (120056939..120108944) | http://www.ncbi.nlm.nih.gov/gene/51778 |
| *NEXN* | 1p31.1 | NG_016625.1 | NC_000001.11 (77888515..77948643) | <https://www.ncbi.nlm.nih.gov/gene/91624> |
| *NFATC1* | 18q23 | NG_029226.1 | NC_000018.10 (79395772..79529323) | <https://www.ncbi.nlm.nih.gov/gene/4772> |
| *NKX2-5* | 5q34 | NG_013340.1 | NC_000005.9 (172659107..172662315, complement) | http://www.ncbi.nlm.nih.gov/gene/1482 |
| *NNT* | 5p12 | NG_032869.1 | NC_000005.10 (43601092..43705566) | <https://www.ncbi.nlm.nih.gov/gene/23530> |
| *NR0B1* | Xp21.2 | NG_009814.1 | NC_000023.11 (30304422..30309378, complement) | <https://www.ncbi.nlm.nih.gov/gene/190> |
| *NRAS* | 1p13.2 | NG_007572.1 | NC_000001.10 (115247085..115259515, complement) | http://www.ncbi.nlm.nih.gov/gene/4893 |
| *NRG1* | 8p12 | NG_012005.2 | NC_000008.11 (31639222..32771716) | <https://www.ncbi.nlm.nih.gov/gene/3084> |
| *NSD1* | 5q35.3 | NG_009821.1 | NC_000005.10 (177131835..177300213) | <https://www.ncbi.nlm.nih.gov/gene/64324> |
| *NUMB* | 14q24.2-q24.3 | NG_029061.2 | NC_000014.9 (73275210..73458580, complement) | <https://www.ncbi.nlm.nih.gov/gene/8650> |
| *NUMBL* | 19q13.2 | NC_000019.10 | NC_000019.10 (40665905..40690658, complement) | <https://www.ncbi.nlm.nih.gov/gene/9253> |
| *PKP2* | 12p11 | NG_009000.1 | NC_000012.11 (32943680..33049780, complement) | http://www.ncbi.nlm.nih.gov/gene/5318 |
| *PLEC* | 8q24.3 | NG_012492.1 | NC_000008.11 (143915147..143976800, complement) | <https://www.ncbi.nlm.nih.gov/gene/5339> |
| *PLEKHM2* | 1p36.21 | NG_053033.1 | NC_000001.11 (15681506..15734769) | <https://www.ncbi.nlm.nih.gov/gene/23207> |
| *PLN* | 6q22.1 | NG_009082.1 | NC_000006.11 (118869442..118881587) | http://www.ncbi.nlm.nih.gov/gene/5350 |
| *PMP22* | 17p12 | NG_007949.1 | NC_000017.11 (15229777..15265373, complement) | <https://www.ncbi.nlm.nih.gov/gene/5376> |
| *POLG* | 15q26.1 | NG_008218.2 | NC_000015.10 (89316305..89334795, complement) | <https://www.ncbi.nlm.nih.gov/gene/5428> |
| *PRDM16* | 1p36.32 | NG_029576.1 | NC_000001.11 (3068227..3438621) | <https://www.ncbi.nlm.nih.gov/gene/63976> |
| *PRKAG2* | 7q36.1 | NG_007486.1 | NC_000007.13 (151253200..151574316, complement) | http://www.ncbi.nlm.nih.gov/gene/51422 |
| *PTGS2* | 1q31.1 | NG_028206.2 | NC_000001.11 (186671812..186680427, complement) | <https://www.ncbi.nlm.nih.gov/gene/5743> |
| *PTK2* | 8q24.3 | NG_029467.1 | NC_000008.11 (140658382..141002079, complement) | <https://www.ncbi.nlm.nih.gov/gene/5747> |
| *PTPN11* | 12q24 | NG_007459.1 | NC_000012.11 (112856536..112947717) | http://www.ncbi.nlm.nih.gov/gene/5781 |
| *RAD54L2* | 3p21.2 | NC_000003.12 | NC_000003.12 (51538683..51668660) | <https://www.ncbi.nlm.nih.gov/gene/23132> |
| *RAF1* | 3p25 | NG_007467.1 | NC_000003.11 (12625100..12705700, complement) | http://www.ncbi.nlm.nih.gov/gene/5894 |
| *RANGRF* | 17p13.1 | NG_028189.1 | NC_000017.11 (8288497..8290092) | <https://www.ncbi.nlm.nih.gov/gene/29098> |
| *RBM20* | 10q25.2 | NG_021177.1 | NC_000010.11 (110641933..110839471) | <https://www.ncbi.nlm.nih.gov/gene/282996> |
| *RIT1* | 1q22 | NG_033885.1 | NC_000001.11 (155897808..155911402, complement) | <https://www.ncbi.nlm.nih.gov/gene/6016> |
| *RPS6KA3* | Xp22.12 | NG_007488.1 | NC_000023.11 (20149911..20267514, complement) | <https://www.ncbi.nlm.nih.gov/gene/6197> |
| *RPS7* | 2p25 | NG_011744.1 | NC_000002.11 (3622853..3628509) | http://www.ncbi.nlm.nih.gov/gene/6201 |
| *RYR1* | 19q13.2 | NG_008866.1 | NC_000019.10 (38433700..38587564) | <https://www.ncbi.nlm.nih.gov/gene/6261> |
| *RYR2* | 1q43 | NG_008799.2 | NC_000001.10 (237205510..237997288) | http://www.ncbi.nlm.nih.gov/gene/6262 |
| *SCN1B* | 9q13.1 | NG_013359.1 | NC_000019.9 (35521555..35531353) | http://www.ncbi.nlm.nih.gov/gene/6324 |
| *SCN3B* | 11q23.3 | NG_016283.1 | NC_000011.9 (123499895..123525315, complement) | http://www.ncbi.nlm.nih.gov/gene/55800 |
| *SCN4B* | 11q23.3 | NG_011710.1 | NC_000011.9 (118004092..118023630, complement) | http://www.ncbi.nlm.nih.gov/gene/6330 |
| *SCN5A* | 3p21 | NG_008934.1 | NC_000003.11 (38589553..38691164, complement) | http://www.ncbi.nlm.nih.gov/gene/6331 |
| *SCO2* | 22q13.33 | NG_016235.1 | NC_000022.11 (50523568..50526439, complement) | <https://www.ncbi.nlm.nih.gov/gene/9997> |
| *SCRIB* | 8q24.3 | NG_030583.1 | NC_000008.11 (143790920..143815379, complement) | <https://www.ncbi.nlm.nih.gov/gene/23513> |
| *SDHA* | 5p15.33 | NG_012339.1 | NC_000005.10 (218223..264816) | <https://www.ncbi.nlm.nih.gov/gene/6389> |
| *SDHAF1* | 19q13.12 | NG_016869.1 | NC_000019.10 (35995188..35996318) | <https://www.ncbi.nlm.nih.gov/gene/644096> |
| *SDHB* | 1p36.13 | NG_012340.1 | NC_000001.11 (17018722..17054170, complement) | <https://www.ncbi.nlm.nih.gov/gene/6390> |
| *SDS* | 12q24.13 | NC_000012.12 | NC_000012.12 (113392445..113403887, complement) | <https://www.ncbi.nlm.nih.gov/gene/10993> |
| *SGCD* | 5q33-q34 | NG_008693.2 | NC_000005.9 (155462147..156194799) | http://www.ncbi.nlm.nih.gov/gene/6444 |
| *SLC22A5* | 5q31.1 | NG_008982.2 | NC_000005.10 (132369704..132395614) | https://www.ncbi.nlm.nih.gov/gene/6584 |
| *SLC25A20* | 3p21.31 | NG_008171.1 | NC_000003.12 (48856923..48898993, complement) | https://www.ncbi.nlm.nih.gov/gene/788 |
| *SLC25A4* | 4q35 | NG_013001.1 | NC_000004.11 (186064417..186071538) | http://www.ncbi.nlm.nih.gov/gene/291 |
| *SLC25A4* | 4q35.1 | NG_013001.1 | NC_000004.12 (185143263..185150384) | https://www.ncbi.nlm.nih.gov/gene/291 |
| *SLC25A5* | Xq24 | NG_013262.1 | NC_000023.11 (119468400..119471396) | <https://www.ncbi.nlm.nih.gov/gene/292> |
| *SLC52A2* | 8q24.3 | NG_032872.1 | NC_000008.11 (144358547..144361286) | <https://www.ncbi.nlm.nih.gov/gene/79581> |
| *SMAD3* | 15q22.33 | NG_011990.1 | NC_000015.9 (67358036..67487533) | http://www.ncbi.nlm.nih.gov/gene/4088 |
| *SMAD7* | 18q21.1 | NG_023330.1 | NC_000018.10 (48919853..48950711, complement) | <https://www.ncbi.nlm.nih.gov/gene/4092> |
| *SMARCA4* | 19p13.2 | NG_011556.2 | NC_000019.10 (10960922..11062282) | <https://www.ncbi.nlm.nih.gov/gene/6597> |
| *SNTA1* | 20q11.2 | NG_011622.1 | NC_000020.10 (31995763..32031698, complement) | http://www.ncbi.nlm.nih.gov/gene/6640 |
| *SOS1* | 2p21 | NG_007530.1 | NC_000002.11 (39208690..39347686, complement) | http://www.ncbi.nlm.nih.gov/gene/6654 |
| *SQSTM1* | 5q35.3 | NG_011342.1 | NC_000005.10 (179806388..179838078) | <https://www.ncbi.nlm.nih.gov/gene/8878> |
| *STARD3* | 17q11-q12 |  | NC_000017.10 (37793333..37820454) | http://www.ncbi.nlm.nih.gov/gene/10948 |
| *TAZ* | Xq28 | NG_009634.1 | NC_000023.10 (153639877..153650065) | http://www.ncbi.nlm.nih.gov/gene/6901 |
| *TBX20* | 7p14.2 | NG_015805.1 | NC_000007.14 (35199936..35254100, complement) | <https://www.ncbi.nlm.nih.gov/gene/57057> |
| *TBX5* | 12q24.1 | NG_007373.1 | NC_000012.11 (114791735..114846247, complement) | http://www.ncbi.nlm.nih.gov/gene/6910 |
| *TCAP* | 17q12 | NG_008892.1 | NC_000017.11 (39665346..39666554) | <https://www.ncbi.nlm.nih.gov/gene/8557> |
| *TGFB3* | 14q24.3 | NG_011715.1 | NC_000014.9 (75958061..75983011, complement) | <https://www.ncbi.nlm.nih.gov/gene/7043> |
| *TGFBR1* | 9q22 | NG_007461.1 | NC_000009.11 (101867412..101916474) | http://www.ncbi.nlm.nih.gov/gene/7046 |
| *TGFBR2* | 3p22 | NG_007490.1 | NC_000003.11 (30647994..30735634) | http://www.ncbi.nlm.nih.gov/gene/7048 |
| *TMEM43* | 3p25.1 | NG_008975.1 | NC_000003.11 (14166440..14185180) | http://www.ncbi.nlm.nih.gov/gene/79188 |
| *TMEM70* | 8q21.11 | NG_016618.1 | NC_000008.11 (73976142..73982783) | <https://www.ncbi.nlm.nih.gov/gene/54968> |
| *TNNC1* | 3p21.1 | NG_008963.1 | NC_000003.11 (52485107..52488057, complement) | http://www.ncbi.nlm.nih.gov/gene/7134 |
| *TNNI3* | 19q13.4 | NG_007866.2 | NC_000019.9 (55663135..55669100, complement) | http://www.ncbi.nlm.nih.gov/gene/7137 |
| *TNNT2* | 1q32 | NG_007556.1 | NC_000001.10 (201328136..201346836, complement) | http://www.ncbi.nlm.nih.gov/gene/7139 |
| *TPM1* | 15q22.1 | NG_007557.1 | NC_000015.9 (63334838..63364114) | http://www.ncbi.nlm.nih.gov/gene/7168 |
| *TTN* | 2q31.2 | NG_011618.3 | NC_000002.12 (178525989..178807423, complement) | <https://www.ncbi.nlm.nih.gov/gene/7273> |
| *TTR* | 18q12.1 | NG_009490.1 | NC_000018.10 (31591767..31599024) | <https://www.ncbi.nlm.nih.gov/gene/7276> |
| *VANGL2* | 1q23.2 | NG_023420.1 | NC_000001.11 (160400574..160428678) | <https://www.ncbi.nlm.nih.gov/gene/57216> |
| *VCL* | 10q22.2 | NG_008868.1 | NC_000010.10 (75757836..75879918) | http://www.ncbi.nlm.nih.gov/gene/7414 |
| *YAP1* | 11q22.1 | NG_029530.1 | NC_000011.10 (102109957..102233423) | <https://www.ncbi.nlm.nih.gov/gene/10413> |
| *YWHAE* | 17p13.3 | NG_009233.1 | NC_000017.11 (1344539..1400262, complement) | <https://www.ncbi.nlm.nih.gov/gene/7531> |
| *ZNF25* | 10p11.21 | NC_000010.11 | NC_000010.11 (37949572..37976655, complement) | <https://www.ncbi.nlm.nih.gov/gene/219749> |

**Table S2. Silico predictive algorithms used in the study.**

| Category | Basis | Name | Website | Prediction Threshold |
| --- | --- | --- | --- | --- |
| Missense prediction | Evolutionary conservation | FATHMM | http://fathmm.biocompute.org.uk | <-1.5 Damaging  >-1.5 Tolerated |
|  |  | SIFT | http://sift.jcvi.org | <0.05 Deleterious  >0.05 Tolerated |
| Missense prediction | Protein structure/function and evolutionary conservation | Align GVGD | http://agvgd.iarc.fr/agvgd_input.php | ≧C15 Probably Damaging |
|  |  | Mutation Taster | http://www.mutationtaster.org | Disease causing |
|  |  | Polyphen-2 | http://genetics.bwh.harvard.edu/pph2 | 0.85 to 1 Probably Damage  0.15 to 0.85 Possibly Damage |
| Missense and insertion/deletions  prediction | Alignment and measurement of similarity between variant sequence and protein sequence homolog | PROVEAN | http://provean.jcvi.org/index.php | <-2.5 Deleterious  >-2.5 Neutral |
| Missense and insertion/deletions  prediction | Contrasts annotations of fixed/nearly fixed derived alleles in humans with simulated variants | CADD | http://cadd.gs.washington.edu | ≧20 1% most deleterious  ≧30 0.1%most deleterious |

Reference

1.Richards S, Aziz N, Bale S, Bick D, Das S, Gastier-Foster J, Grody WW, Hegde M, Lyon E, Spector E, Voelkerding K, Rehm HL. Standards and guidelines for the interpretation of sequence variants: a joint consensus recommendation of the American College of Medical Genetics and Genomics and the association for molecular autopsy. Genet Med 2015;17:405–423.
